# Neuropathy of the infraorbital area following rapid palatal expansion: A case report

**DOI:** 10.1097/MD.0000000000047055

**Published:** 2026-01-09

**Authors:** Sung Min Kim, Hong-Seop Kho

**Affiliations:** a Department of Oral Medicine and Oral Diagnosis, School of Dentistry and Dental Research Institute, Seoul National University, Seoul, Republic of Korea; b Institute on Aging, Seoul National University, Seoul, Republic of Korea.

**Keywords:** case report, gabapentin, hypoesthesia, palatal expansion technique, trigeminal nerve neuropathy

## Abstract

**Rationale:**

Neuropathic pain in the orofacial region, although relatively uncommon, can occur after dental procedures performed in trigeminally innervated areas. Miniscrew-assisted rapid palatal expansion (MARPE) is widely adopted to correct maxillary transverse deficiency in adults and is generally safe; however, neurosensory complications are rarely documented. This case highlights infraorbital neuropathy following MARPE, underscoring the need for clinical vigilance.

**Patient concerns:**

A healthy 28-year-old female presented with numbness and tenderness on the left midface approximately 5 weeks after MARPE appliance placement. Her symptoms included hypesthesia in the zygomatic, upper lip, philtral, and palatal regions, and palpation-induced pain in the buccal mucosa and lower lip. The discomfort was described as dull and throbbing, partially numb, provoked by touch but not by thermal stimuli, with a visual analog scale score of 2 to 3.

**Diagnoses:**

Clinical examination revealed normal oral function and painless maximal opening. Sensory testing demonstrated marked asymmetry: reduced direction discrimination (30% vs 100%), abnormal 2-point discrimination (perceiving 1 point as 2), elevated pressure pain threshold (60 g vs 30 g), and diminished cold perception, while contact threshold remained symmetrical. Standard panoramic and Waters’ radiographs showed no bony abnormalities. A provisional diagnosis of left maxillary trigeminal neuropathy, localized to the infraorbital nerve distribution, was made.

**Interventions:**

Pharmacological treatment was initiated with gabapentin titrated to 300 mg 3 times daily, a short course of oral prednisolone, and subsequent introduction of low-dose amitriptyline for enhanced neuropathic pain control. Medication was well tolerated except for mild oral dryness and drowsiness, which resolved with tapering.

**Outcomes:**

Serial follow-up over 5 months demonstrated progressive reduction in neurosensory deficits, with near-complete resolution by the third month and full recovery without recurrence at the fifth month.

**Lessons:**

This case illustrates that MARPE, while effective and generally safe, can occasionally induce neuropathic complications extending beyond palatal branches to involve the infraorbital pathway. Mechanical stress redistribution in skeletally mature patients likely contributes to this outcome. Careful monitoring for atypical sensory changes, early recognition, and timely pharmacological intervention are essential to optimize prognosis. Further research is warranted to clarify biomechanical mechanisms and risk factors for trigeminal nerve injury during orthodontic maxillary expansion.

## 1. Introduction

Neuropathic pain is defined as pain resulting from a lesion or disease affecting the peripheral or central somatosensory nervous system. It often presents with spontaneous or evoked symptoms, such as burning, shooting, or electric shock-like sensations, and may be accompanied by paresthesia or dysesthesia.^[1,2]^ In the orofacial region, neuropathic pain is commonly associated with post-traumatic trigeminal neuropathy, which may follow dental procedures, including tooth extraction, endodontic therapy, implant placement, orthognathic surgery, or local anesthetic injection.^[1,3,4]^ Its variable presentation, overlap with other chronic orofacial pain conditions, and frequent resistance to treatment make neuropathic orofacial pain a significant diagnostic and therapeutic challenge.^[5,6]^

Rapid palatal expansion (RPE) is an orthodontic technique designed to correct maxillary transverse deficiency by opening the midpalatal suture.^[7]^ While traditionally reserved for growing patients, RPE has been adapted for adults through surgical assistance or skeletal anchorage.^[8,9]^ Miniscrew-assisted rapid palatal expansion (MARPE) enables skeletal expansion without osteotomy, providing a less invasive alternative with reduced morbidity.^[10,11]^ Although generally safe, RPE has rarely been associated with neurosensory complications, including paresthesia, tinnitus, and headache.^[12–14]^ This case report describes a rare instance of unilateral infraorbital neuropathic symptoms in a healthy female adult following MARPE. Comprehensive neurosensory assessment confirmed localized hypesthesia, which resolved completely after pharmacological management.

## 2. Case presentation

**Figure 1.**
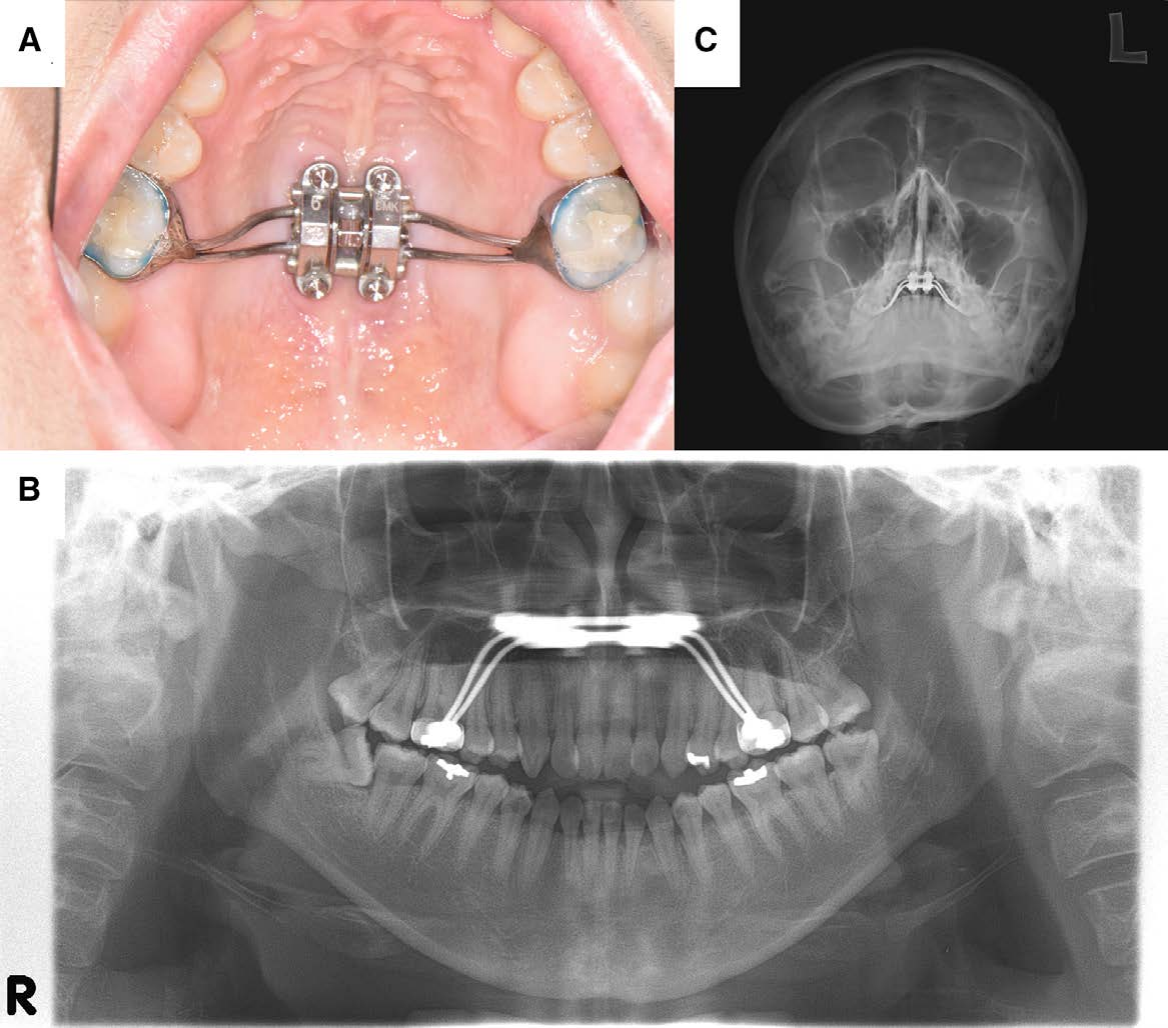
(A) Intraoral photograph showing a MARPE appliance. (B) Standard panoramic radiograph revealing the MARPE appliance with no detectable bony abnormalities or asymmetry. (C) Radiographs obtained from the Waters’ view showing clear paranasal sinus outlines and the absence of midfacial skeletal asymmetry. MARPE = miniscrew-assisted rapid palatal expansion.

The Institutional Review Board (IRB) of Seoul National University Dental Hospital approved this study (IRB No.: ERI25043). The IRB also exempted the requirement for obtaining informed consent, as all clinical data and images were fully anonymized and used retrospectively in accordance with ethical standards. A 28-year-old female patient presented to the Department of Oral Medicine with unilateral facial numbness and tenderness, predominantly involving the left midface and oral region. She reported that a MARPE appliance (Fig. 1A) had been placed at an external dental clinic approximately 6 weeks before her initial visit and activated at 2 turns (approximately 0.27 mm) per day. Approximately 5 weeks after appliance placement, she developed sensory disturbances in the left infraorbital region. According to the patient, the direction of activation was reversed at the same rate approximately 1 week later, which led to gradual symptom improvement. She noted partial symptom relief in the palate and upper lip, whereas hypesthesia persisted in the left philtrum and zygomatic area, accompanied by palpation-induced pain in the left buccal mucosa and lower lip. The total expansion amount and screw specifications could not be verified because the appliance was fabricated and adjusted externally.

**Figure 2.**
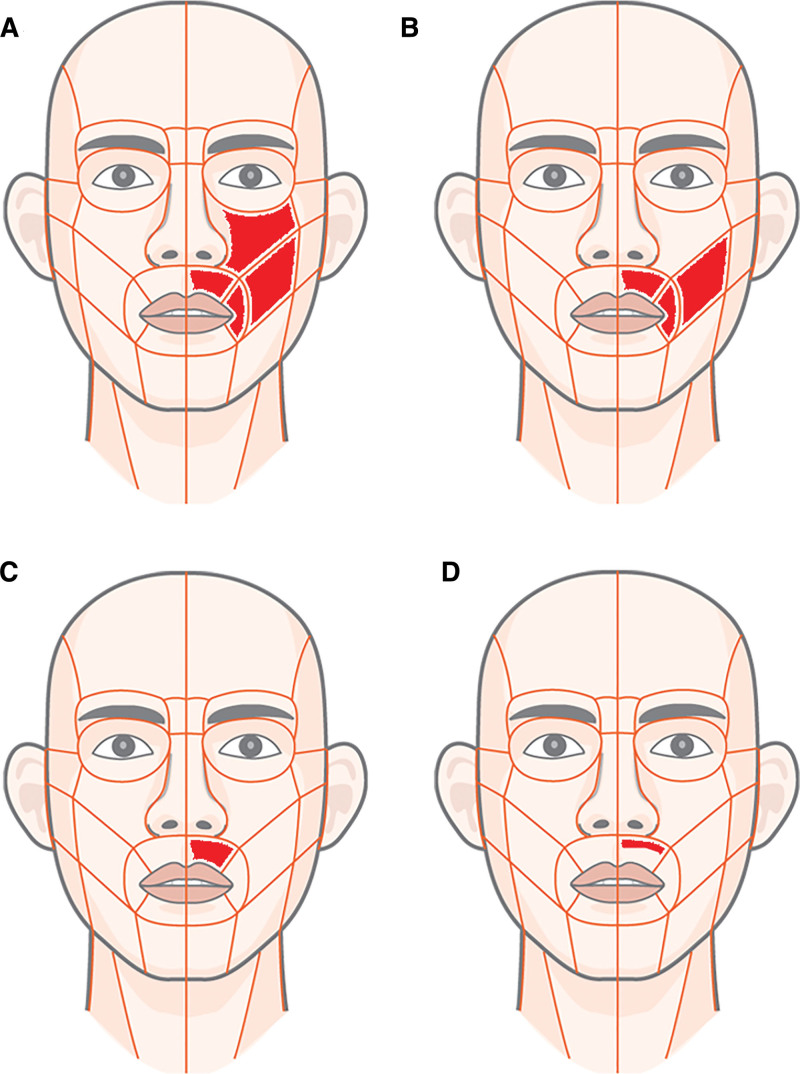
Serial changes in the distribution of sensory symptoms in the left midfacial region. Red-shaded areas indicate regions of patient reported sensory disturbances. (A) At the initial visit, hypesthesia and tenderness were present in the left zygomatic, buccal, philtral, and palatal areas. (B) 3 wk later, zygomatic pain had improved, but philtral hypesthesia and mild cheek tenderness persisted. (C) At 6 wk, zygomatic and buccal symptoms had almost completely resolved, but residual philtral hypoesthesia was observed. (D) At 9 wk, only minimal philtral discomfort during lip movement remained.

At the initial visit, the patient reported numbness in the left zygomatic region, upper lip, philtrum, and palate, accompanied by palpation-induced pain in the left buccal mucosa and lower lip (Fig. 2A). The discomfort was described as dull and throbbing with partial numbness, triggered by touch but not by thermal stimuli and rated 2 to 3 on the visual analog scale (VAS). The patient denied spontaneous pain, trauma history, nocturnal awakening due to pain, or prior relief from nonsteroidal anti-inflammatory drugs. Medical, family, and psychosocial histories were unremarkable.

Clinical examination revealed normal oral function, with a painless maximum mouth opening of 55 mm. Palpation of the temporomandibular joints and masticatory muscles was non-tender bilaterally, and no facial asymmetry or muscle weakness was observed. Standard panoramic and Waters’ view radiographs revealed no bony abnormalities or asymmetry (Fig. 1B and C), although possible skeletal displacement could not be assessed due to the absence of pre-expansion imaging. No additional cone beam computed tomography (CBCT) imaging was performed after symptom onset because the patient showed gradual sensory improvement following reversal of appliance activation and pharmacologic therapy. As no surgical intervention was required, further radiographic exposure was avoided in accordance with the as low as reasonably achievable principle. No financial or cultural barriers affected diagnosis, but the absence of pre-expansion imaging posed a diagnostic challenge in assessing skeletal changes.

Quantitative sensory testing (QST) was performed using a standardized orofacial neurosensory testing protocol, including contact threshold (CTH), direction discrimination (DIR), 2-point discrimination, pin-prick (PP), and cold perception assessments. All tests were conducted by a single trained examiner in a quiet environment under consistent conditions.

CTH and DIR were measured using calibrated von Frey filaments (North Coast Medical, Gilroy; No. 1.65–6.65), applied perpendicularly for approximately 1 second until slight bending occurred. DIR was evaluated with 20 randomized strokes (right, left, or downward) and expressed as the percentage of correct responses. 2PD was tested using a mechanical compass with rounded 23-gauge needle tips and a millimeter scale, and PP thresholds were measured with a calibrated dial tension gauge (TECLOCK Co., LTD., Okaya, Nagano, Japan) with a rounded needle tip, increasing by 10 g increments up to 150 g. Cold sensory testing was conducted by lightly applying a cotton swab soaked in freezer-kept ethanol to the test site, with the patient asked to report the perception of coolness and any side-to-side differences.

Each test was repeated 3 times per site, and mean values were recorded. Reliability was maintained using an ascending–descending sequence to minimize examiner bias. Reference normative data for orofacial regions were based on previously established sensory testing reference, indicating average CTH values of No. 1.65 ± 0.00, DIR accuracy of 92.75 ± 8.18%, 2PD thresholds of 9.15 ± 1.56 mm, and PP thresholds of 58.20 ± 12.62 g in healthy female adults.^[15]^

Compared with the contralateral side, the patient showed marked sensory asymmetry in the distribution of the left infraorbital nerve (Table 1). DIR accuracy was 30% (6/20) on the left versus 100% (20/20) on the right. 2PD was abnormally reduced on the left, where a single point was perceived as 2, while the right side showed a normal threshold of 10 mm. PP threshold was elevated on the left (60 g) compared with the right (30 g), indicating hypesthesia. Cold perception was reduced on the left, whereas CTH testing showed no significant asymmetry.

**Table 1:** Summary of QST results for both sides of the infraorbital region.

	Right	Left
DIR	100% (20/20)	30% (6/20)
2PD	10 mm	Undetectable
PP	30 g	60 g
Thermal	Cold	Less cold

QST was performed to assess tactile and thermal functions in the infraorbital region.

DIR was evaluated by asking the patient to identify the direction of 20 randomized strokes applied with von Frey filaments. 2PD was measured using a mechanical compass to determine the minimal distance at which 2 points were perceived as distinct. Pressure pain threshold was determined using a dial tension gauge with a rounded needle tip, increasing pressure in 10 g increments until pain was perceived. Thermal perception was assessed by lightly applying a cotton swab soaked in ethanol cooled in a freezer to evaluate the sensation of cold.

2PD = 2-point discrimination, DIR = direction discrimination, PP = pin-prick test, QST = quantitative sensory testing.

Other possible causes of facial numbness were clinically excluded. Local anesthesia had been administered for MARPE placement several weeks before symptom onset; however, the injection site was distant from the infraorbital region, and no immediate postoperative sensory changes were reported. The patient had no history or symptoms suggestive of systemic metabolic disorders such as diabetes or vitamin B_12_ deficiency. Intraoral and radiographic examinations revealed no evidence of infection, osteitis, or sinus pathology. Temporomandibular joint and masticatory muscle findings were normal, and neurosensory testing showed sensory deficits limited to the infraorbital distribution, consistent with infraorbital nerve neuropathy.

Based on these clinical findings, a provisional diagnosis of left maxillary nerve neuropathy was made. Pharmacological treatment was initiated with 300 mg of gabapentin once daily, which was titrated to 3 times daily over 7 days. To address potential inflammation, prednisolone (20 mg/day) was prescribed for 5 days.

Approximately 1 week after the initial visit, the patient reported partial symptom improvement, particularly in the zygomatic area. Philtral hypesthesia persisted, but palpation-induced discomfort in the buccal mucosa and lower lip had lessened, with the VAS score decreased to 1 to 2. Gabapentin (300 mg TID) was continued, and prednisolone was discontinued without adverse events apart from mild oral dryness. At 3 weeks, the dull zygomatic pain had further improved. Philtral hypesthesia and mild tenderness remained upon cheek tapping (Fig. 2B). Gabapentin (300 mg TID) administration continued, and amitriptyline (10 mg) was administered at bedtime. No significant side effects were observed.

Six weeks after the initial visit, zygomatic and buccal symptoms were nearly resolved (VAS 1), although philtral hypesthesia persisted (Fig. 2C). Gabapentin (300 mg TID) was continued, and amitriptyline was increased to 10 mg BID. The patient reported no adverse effects. By 9 weeks, only vague philtral discomfort during lip movement remained (Fig. 2D; VAS 0.5). Gabapentin and amitriptyline administration continued at the same doses. The patient experienced mild drowsiness and medication tapering was planned.

**Figure 3.**
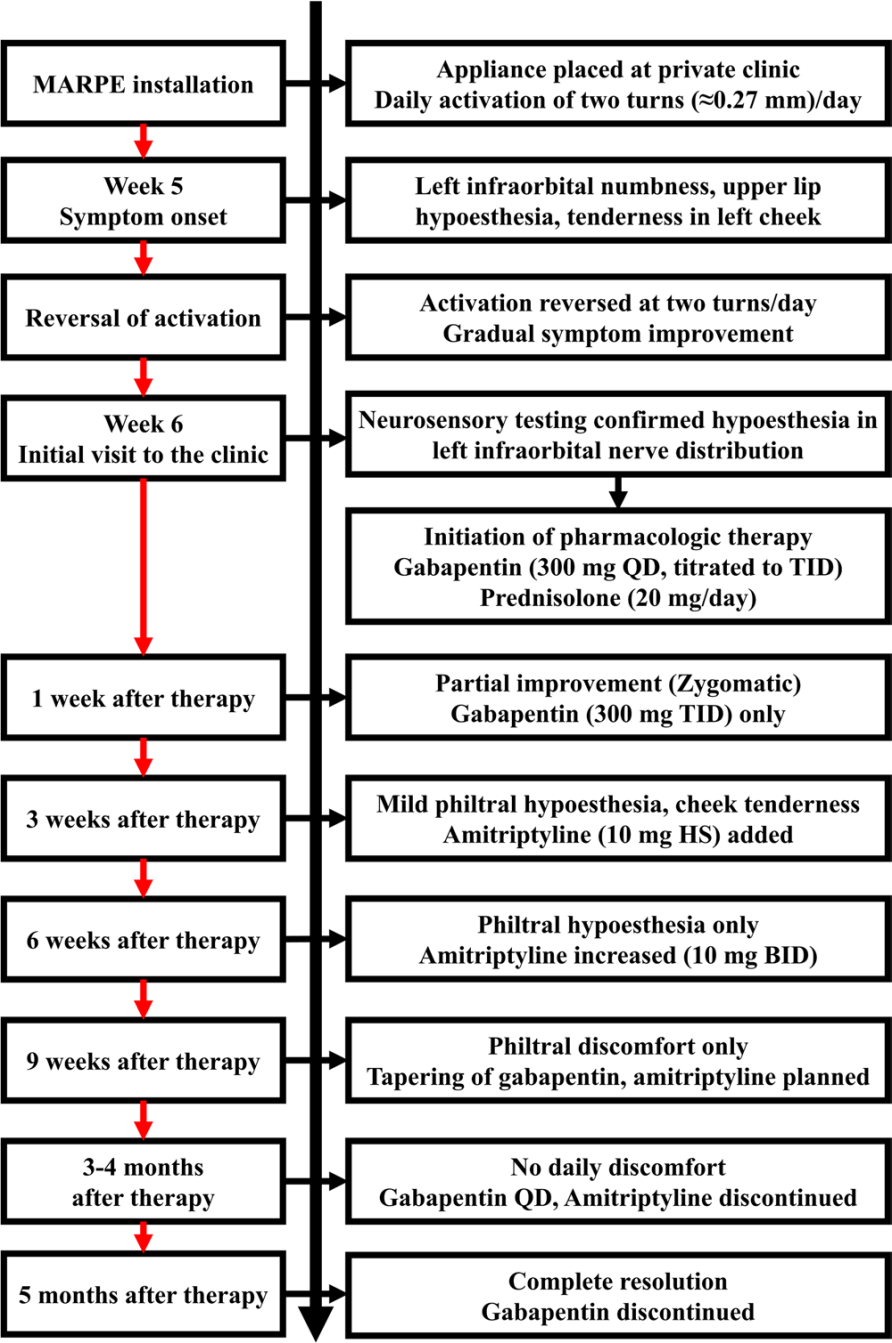
Clinical timeline of the patient from MARPE appliance placement to complete recovery. Symptom progression and therapeutic milestones are displayed to illustrate the temporal relationship between appliance activation, intervention, and resolution of neuropathic symptoms. BID = twice daily, HS = before bedtime, MARPE = miniscrew-assisted rapid palatal expansion, QD = once daily, TID = 3 times daily.

At 3 and 4 months, the symptoms had nearly resolved, with no daily discomfort. Amitriptyline was tapered to once daily at 3 months and discontinued by 4 months; gabapentin was tapered to once daily. At the final follow-up visit, 5 months after the initial presentation, the patient reported complete resolution of the neurosensory symptoms without tenderness or paresthesia. Pharmacologic therapy was maintained for approximately 5 months in total, resulting in complete resolution of symptoms without recurrence. Gabapentin was discontinued and long-term follow-up was scheduled. The chronological course of the patient, including appliance placement, activation, onset of symptoms, pharmacologic management, and recovery, is summarized in Figure 3.

The patient showed good adherence to the prescribed medication and scheduled follow-up visits. No significant discomfort or interference with daily activities was reported during the observation period. The treatment was completed uneventfully, and the patient’s condition fully recovered without residual symptoms.

## 3. Discussion

This case illustrates a rare but clinically significant complication of MARPE in the form of unilateral infraorbital neuropathic symptoms. While most patients experience transient discomfort localized to the palatal branches of the maxillary nerve,^[14,16]^ the numbness and dysesthesia observed here suggest infraorbital nerve involvement.^[12,13]^ Other potential causes of facial numbness, including local anesthetic trauma, infection, metabolic disease, and temporomandibular disorders, were clinically excluded. The several-week delay between appliance placement and symptom onset made anesthetic-induced neuropathy unlikely, suggesting that gradual mechanical stress or late-phase displacement may have contributed.

The infraorbital nerve, a terminal branch of the maxillary division of the trigeminal nerve, supplies sensation to the lower eyelid, upper lip, lateral nose, and upper cheek.^[17]^ Transversing the infraorbital canal and emerging through the thin anterior maxillary wall, it is susceptible to injury from midfacial trauma or surgical manipulation.^[18,19]^ Studies have shown that MARPE-generated forces extend beyond the midpalatal suture to multiple craniofacial structures, including the zygomaticomaxillary and pterygopalatine sutures.^[7,11,20]^

In skeletally mature individuals, greater suture interdigitation and ossification increase resistance to expansion, redirecting mechanical stress towards the cranial base and areas innervated by maxillary nerve branches.^[19,21,22]^ This redistribution may increase the mechanical loading within the infraorbital region, increasing the risk of compressive or traction-related neuropathy. Finite element models have demonstrated that stress concentrations during RPE are particularly pronounced around key neurovascular foramina, especially in adult bone with reduced elasticity.^[23,24]^ The zygomaticomaxillary suture has been identified as a major site of resistance during maxillary expansion, further supporting the likelihood of localized stress amplification.^[25,26]^ Recent finite element analyses of MARPE have similarly shown that mechanical stresses extend toward the infrazygomatic and infraorbital regions, suggesting potential transmission of force to the infraorbital canal.^[27]^ Even without gross skeletal displacement, these transmitted forces may alter cranial base dynamics and compromise adjacent neurovascular structures, underscoring the potential for subclinical nerve injury in anatomically predisposed individuals.

In this case, neurosensory testing confirmed significant hypesthesia in the infraorbital nerve distribution, with reduced tactile and thermal sensation, impaired 2PD, and elevated pain thresholds. These findings suggest involvement of Aβ fibers (tactile sensation), Aδ fibers (cold and sharp pain), and possibly C fibers. While mild reduction in cool sensation was observed, noxious cold and heat thresholds were not assessed, limiting definitive conclusions regarding C fiber function.^[28,29]^

Experimental and clinical studies suggest that Aβ fibers are particularly susceptible to compression or stretch-related injury, potentially explaining the observed tactile deficits.^[28,30]^ The absence of radiographic abnormalities does not preclude nerve dysfunction, underscoring the value of QST for the early detection of trigeminal neuropathy, even when imaging is inconclusive.

The clinical course in this case suggests that early reversal of the expansion forces, combined with prompt neuropathic pain management, may be effective. Pharmacological treatment with gabapentin and low-dose amitriptyline, both established agents for neuropathic pain, facilitated progressive symptom resolution over several months, consistent with a reversible neuropathic process – likely involving neuropraxic nerve injury rather than permanent axonal disruption.^[31]^ The combination of gabapentin, a calcium channel α2δ subunit modulator, and amitriptyline, a tricyclic antidepressant enhancing noradrenergic and serotonergic inhibition, is widely recognized to provide synergistic analgesia and improved tolerability compared with monotherapy in chronic neuropathic pain conditions.^[32]^

This case report has certain limitations. Pre-expansion CBCT or panoramic imaging was unavailable because the MARPE procedure had been performed at an external dental clinic prior to referral. Consequently, direct comparison of skeletal displacement or infraorbital canal morphology before and after expansion was not possible. No post-onset CBCT imaging was obtained, as the patient demonstrated progressive recovery and further exposure was avoided following the as low as reasonably achievable principle. Furthermore, the absence of detailed technical specifications of the expansion device limited the precision of mechanical correlation analysis. Despite these constraints, the temporal sequence of activation, symptom onset, and sensory recovery still provides valuable insight into the potential mechanisms of infraorbital neuropathy associated with MARPE. Additional limitations include the single-case design, lack of electrophysiologic assessments such as electromyography or nerve conduction studies, and a relatively short follow-up period.

Given the anatomical complexity of the infraorbital region, interdisciplinary management may be indicated in cases of persistent or progressive sensory disturbances. Collaboration with neurology, orofacial pain, and otolaryngology specialists can aid in differential diagnosis and comprehensive evaluation of nerve function. Surgical decompression or local nerve block may be considered if symptoms persist beyond several months, worsen despite pharmacologic therapy, or if imaging reveals mechanical compression or entrapment of the infraorbital canal.^[33,34]^

Reports of MARPE-related neuropathies are limited, making this case an important contribution to literature. Although the incidence appears low, clinicians should maintain vigilance for atypical sensory complaints during MARPE, particularly in adults. Comprehensive neurosensory assessments, including QST, are vital for early diagnosis, differential diagnosis, and therapeutic intervention guidance. In clinical practice, careful monitoring of sensory changes during expansion is advised, especially in patients with increased sutural interdigitation or reduced bone elasticity. A slower activation rate and preoperative evaluation of sutural maturity may help minimize the risk of transient or permanent nerve injury.

## 4. Conclusions

In conclusion, this case emphasizes the need for individualized monitoring during MARPE, particularly in skeletally mature patients. Heightened awareness of possible infraorbital nerve involvement, coupled with proactive assessment of atypical facial symptoms, can enable timely intervention and improved prognosis. Further research is warranted to elucidate the biomechanical mechanisms, risk factors, and long-term implications of neuropathic complications associated with orthodontic skeletal expansion techniques.

## Author contributions

**Conceptualization:** Sung Min Kim, Hong-Seop Kho.

**Investigation:** Sung Min Kim, Hong-Seop Kho.

**Resources:** Hong-Seop Kho.

**Supervision:** Hong-Seop Kho.

**Visualization:** Sung Min Kim.

**Writing – original draft:** Sung Min Kim, Hong-Seop Kho.

**Writing – review & editing:** Sung Min Kim, Hong-Seop Kho.
